# Research on the intervention mechanism and dynamic evolution of the digital divide’s impact on the physical and mental health of older adults: a system dynamics perspective

**DOI:** 10.3389/fpubh.2026.1838980

**Published:** 2026-05-28

**Authors:** Tianshu Shao, Yixuan Chen, Benyan Ren, Sheng Gang

**Affiliations:** 1 Zhejiang University of Finance and Economics Dongfang College, Haining, China; 2 Jiang Su High Hope Group, Nanjing, China

**Keywords:** digital divide, older adults, physical and mental health, social support, system dynamics

## Abstract

With the trend of global population aging and digital transformation, the problem of the digital divide among older adults has become increasingly serious. Narrowing the digital divide is a necessary way to improve the physical and mental health of older adults. Since the impact of the digital divide on the physical and mental health of older adults is a dynamic process involving feedback effects among multiple factors, this paper constructs a dynamic simulation model from the system dynamics perspective, and including variables such as digital literacy level, positive attitude toward aging, social network support intensity, and overall health and well-being. This study examines the feedback mechanisms by which digital divide affects the physical and mental health of older adults, overcoming the limitation of traditional empirical research in capturing dynamic interactions occurring within the system. The results show that the overall health and well-being of older adults increases 28% over the 100-month simulation period, showing an upward trend at first and then tends to stabilize. Sensitivity analysis indicates that the influence of policy support intensity, intensity of digital reverse mentoring in families, and digital device age-friendly adaptation level on the overall health and well-being of older adults decreases in that order. Based on the results, this paper identifies positive attitude toward aging, social adaptability, and social network support intensity as mediating variables in the empowerment of older adults’ physical and mental health through digital technology, thereby enriching the application of system dynamics in the field of digital health.

## 1 Introduction

The launch of ChatGPT during 2022 signifies the profound penetration of worldwide society into the digital era, as digital tools are extensively utilized throughout various facets of human life. The incorporation of electronic innovations into conventional sectors has emerged as a vital driver propelling global economic expansion. Nevertheless, the advancement of computerized systems has simultaneously introduced the challenge of the digital divide, intensifying the disparity in digital accessibility among the international senior citizen demographic. This creates a further chasm, representing a “communication isolation” of our seniors within the modern digital epoch. Investigations into the effects of this digital divide on the physical and mental of older adults have increasingly transformed into a focal topic within the realm of international public health. Globally, global entities like the World Health Organization and the Pan American Health Organization offer significant concern regarding the worldwide digital divide and assert that this divide further intensifies the overall inequity in global public health across the board. The most serious is the inequality in health equity, which is an important part of the assessment tool. In this global issue, China, as the country with the largest older adult population in the world, is facing the dual challenges of digital transformation and accelerated population aging. This makes the digital divide problem in China particularly complex and severe. As a country with the fastest growth of older adults in the world, the issue of “digital divide” is extremely urgent in our country. Identify the various elements influencing the physical and mental health of older adults. Based on the 2020 Seventh National Population Census in China, individuals 65 years or older represented 13.50%, suggesting that China has entered an aging society. Furthermore, Chinese seniors possess a relatively limited degree of digital literacy, which exacerbates the perceived divide between the aging population and the era of digital evolution, where the digital divide remains highly pronounced. Simultaneously, the government has started prioritizing the digital divide among the aged, and numerous strategies have been launched. On January 11, 2024, the “Opinions of the General Office of the State Council on Developing Silver Economy to Enhance the Well-being of Older Adults” provided comprehensive strategies and blueprints for empowering the silver economy through digital innovations. The “Work Plan for Promoting High-Quality Development of Digital Technology Adaptation for Older Adults” highlighted the importance of advancing age-friendly services to assist older adults in bridging their digital divide. Nevertheless, the digital divide extends beyond merely hindering physical access like hardware availability; it has transitioned from a mere connectivity issue to influencing social integration and the health physical and mental health of older adults. Numerous researchers have examined the influence of the digital divide on the health status of older adults, yielding substantial findings. On the path to acquiring health knowledge, Cohall et al. highlighted that internet usage enables seniors to gain more medical-related insights and data, potentially enhancing their overall health (1). On the cognitive and mental path, Gupta suggested that the digital divide contributes to a deterioration in cognitive functions among older adults, which adversely affects their mental health (2). Related research on social support has found that digital literacy is positively associated with the level of social support perceived by older adults (3), and social support in turn helps improve older adults’ quality of life and promotes their physical and mental health (4). At the macro level, Li et al., utilizing data spanning 2011 to 2020, observed that the digital divide exerts a detrimental effect on healthy aging (5). These approaches are all studied from the perspective of digital technology, with very few scholars incorporating the key element of cyber risk into their research. Meanwhile, existing studies indicate that digital risks such as online fraud and misinformation significantly increase psychological stress and insecurity among older adults (6), yet such risks remain a gap in research on the digital divide and the physical and mental health of older adults. Furthermore, current studies mostly employ static cross-sectional designs that examine relationships at a single point in time, failing to deeply reveal the dynamic causal relationships and feedback processes between variables (7) (Table 1).

**Table 1:** Comparison of existing research pathways on the digital divide’s impact on the physical and mental health of older adults.

Research path	Core mechanism	Literature	Method limitations
Health knowledge acquisition pathways	The internet can provide more health information, thereby improving the physical and mental of older adults	Cohall et al. (1)	Static cross-sectional design, without controlling for reverse causality
Cognitive and mental pathways	The digital divide leads to cognitive decline, thereby negatively impacting the mental health of older adults	Gupta (2)	Single-dimensional perspective, incomplete causal chain verification
Social support pathways	Social support plays a mediating role in digital literacy and the mental health of older adults	Xia and Zhu (3) and Liu et al. (4)	Short vertical intervals, feedback loops not examined
Macro level	The digital divide slows the progress of healthy aging	Li et al. (5)	Static cross-sectional research cannot capture dynamic feedback
Cyber risk perspective	Digital risks such as online fraud and false information increase psychological stress among older adults	Shang et al. (6)	Static cross-sectional research, lacking longitudinal verification

However, there has been no coupling research conducted among the aforementioned pathways, resulting in a fragmented understanding. First, the health knowledge acquisition pathway emphasizes the information tool function of digital technology (1), the cognitive and mental pathway focuses on internal cognitive resources (2), and the social support pathway highlights interpersonal networks. These three pathways independently examine single mediating mechanisms without being placed within a unified framework (3, 4), and the potential inhibitory or amplifying effects among them have not been considered. For example, whether the health information benefits of digital use are offset by the simultaneous increase in online risks has not been thoroughly investigated in existing research (6). Second, although macro-level longitudinal analyses have revealed the temporal association between the digital divide and healthy aging (5), they are limited by regression designs and cannot identify bidirectional causality and feedback loops between variables. When online risks are taken into account, the health effects of digital technology on older adults are more likely to exhibit nonlinear, stage-specific dynamic characteristics, which cannot be captured in static models.

Therefore, there is still a gap in research on how the digital divide’s impact on the physical and mental health of older adults. First, there is a lack of an integrated framework that can simultaneously accommodate multiple pathways such as health knowledge acquisition (1), changes in cognitive function (2), social support (3, 4), and online risks (6), making it impossible to determine how different intermediate factors collectively shape the overall health outcomes of older adults. Second, there is insufficient examination of feedback loops and long-term dynamic evolutionary processes, leaving the systematic and dynamic predictive foundation required for health interventions still very weak. To fill the above gap, this investigation utilizes China as a case study, constructing a system dynamics model to analyze how digital divide impact the physical and mental health of older adults, incorporating digital empowerment, social support, and online risk feedback mechanisms. The research generates the dynamic simulation trajectory for the comprehensive health evolution of older adults population. This work not only enhances the outcome analysis of the digital divide’s impact on the physical and mental health of older adults but also carries significant theoretical value and practical implications for advancing the high-quality progress of the silver economy. Besides, it utilizes feedback loops to fill the gap in long-term dynamic predictions within current health interventions.

## 2 Materials and methods

### 2.1 Core concepts

Digital divide represents a specific type of societal imbalance stemming from the non-uniform allocation of electronic innovations and the disparate assimilation of online data among various populations (8). First proposed in the 1990s, it has since been studied by many scholars from multiple dimensions. Fundamentally, the digital divide is traditionally classified into three tiers: the access gap, the utilization gap, and the impact gap. The initial tier focuses on access, denoting differences in infrastructure growth, financial assets, and various physical environments (9–11). The secondary tier emphasizes utilization, signifying the distance between individuals’ genuine skill in digital tools and existing technical benchmarks (12). The tertiary tier concentrates on outcomes, characterizing the discrepancy between the achievements users attain via digital systems and their anticipated goals (13). Simultaneously, numerous studies have examined the digital divide from perspectives such as consumption structure, social and spatial dimensions, household income, regional economic growth, resource allocation, and family dynamics (14–16). Ucar et al. (17) pointed out that the digital divide can affect the consumption of different resident groups, which is detrimental to the transformation and upgrading of the consumption structure. Holloway (18) investigated the digital divide through social and geographical lenses, seeking to fulfill the digital requirements of marginalized people and diminish their exclusion. Gorski and Clark (19) discovered that this digital divide intensifies financial disparities among city and countryside dwellers. Ayanso et al. (20) and Arora and Sapre (21) demonstrated that the digital divide leads to uneven regional economic growth. Ji et al. concluded that the digital divide influences the allocation of social resources, widening the gap between the rich and the poor (22). Lee and Kanthawala (23) discovered that in households with high intensity of digital reverse mentoring, the digital literacy and depth of digital usage among older adults significantly improve, resulting in a lower digital divide.

Overall health and well-being of older adults is composed of both their physical and mental health. Given the swift evolution of electronic innovation and the rising proportion of older adults, the influence of the digital divide on the physical and mental health of older adults has emerged as a primary focus of social attention. Some scholars argue that, from the perspective of digital transformation, older adults face a significant digital divide, which turns the convenience brought by digital technology into a burden, negatively affecting their physical and mental health rather than having a positive impact (24, 25). At the same time, as age increases, older adults are highly susceptible to physical and mental health issues such as chronic diseases and loneliness (26–28). Digital devices can help older adults engage in social activities, reduce their sense of loneliness, alleviate mental health issues, and maintain a healthy psychological state (29, 30). Secondly, in the context of rapid digital development, older adults need to continuously improve their digital literacy to adapt to societal demands, which positively contributes to both their physical and mental health (31, 32). Furthermore, Nie and Erbring (33) and Kim et al. (34) collectively argue that the Internet, as a societal platform, might improve the social engagement of seniors, with social involvement serving as a vital mediating factor in the influence of digital technology on their physical and mental health. Lifshitz et al. (35) discovered that social networking behaviors are positively linked to the life fulfillment of the aged. Ultimately, the extent and frequency of Internet utilization by older adults are shaped by variables like bodily fitness, psychological state, digital literacy, and age-friendly devices, with their adoption levels notably below those of middle-aged individuals, positioning them in an isolated status (36–39). Consequently, to mitigate the digital divide between the aged and bolster their physical and mental health, it is essential to augment social media assistance, develop age-accessible solutions, and boost motivation for digital education (40–42).

### 2.2 Theoretical review of relationships between key variable

**Figure 1:**
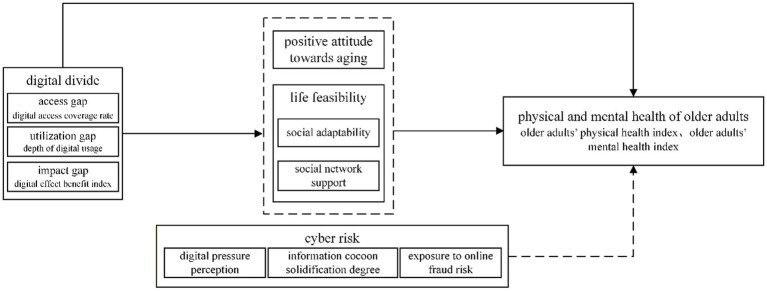
Conceptual framework. Dashed arrows indicate negative impact.

Digital divide is classified as access gaps, utilization gaps, and impact gaps, which exert a substantial detrimental effect on the physical and mental health of older adults (1). The access gap denotes the absence of facilities like electronic connectivity coverage; the usage divide arises from factors like the depth of digital usage; and the outcome divide is the disparity in the digital effect benefit index and related dimensions among different groups in their use of digital technology. An increase in a positive attitude toward aging can reduce loneliness among older adults and enhance their life satisfaction, while a decrease may lead to psychological states such as digital exclusion, thereby affecting their physical and mental health. Subsequently, positive attitude toward aging on senescence functions as an intermediary factor within the process by which the digital divide affects the physical and mental health of older adults (43). Life feasibility includes social adaptability and social network support, which enhance the older adults’ sense of identification with using digital technology and serve as a core pathway for transforming digital technology into improved physical and mental health (44). Consequently, while investigating how the digital divide impacts the physical and mental health of older adults, this research establishes a conceptual framework regarding the digital divide and the physical and mental health of older adults, illustrated in Figure 1.

Additionally, health knowledge acquisition, cognitive and mental, social support, and online risks are not isolated pathways but rather interact and collectively influence each other, forming a complex causal network that determines the overall health and well-being of older adults. Traditional linear regression analysis and static mediation models struggle to capture the bidirectional, time-varying, and self-reinforcing or self-inhibiting dynamic relationships among these variables. To address this limitation, this paper introduces the causal loop diagram, a core analytical tool in system dynamics. In system dynamics, a feedback loop refers to a closed path composed of a series of mutually causal variables. When a change in one variable, through a chain of causal links, ultimately returns to affect itself, a feedback loop is formed. Feedback loops are divided into positive and negative types. Positive feedback loops, where internal changes are continuously amplified, driving the system toward sustained growth or decline; and negative feedback loops, where internal offset mechanisms exist, allowing the system to seek stability and approach a target. The process by which the digital divide among older adults affects their physical and mental health is essentially a dynamic game of trade-offs between the health gains brought by digital empowerment and the psychological losses caused by online risks. Based on the conceptual model in Figure 1, and integrating discussions on core conceptual dimensions such as the digital divide and physical and mental health, this paper synthesizes six core feedback loops that drive system evolution. These feedback loops integrate scattered theoretical pathways into an interconnected, closed-loop dynamic structure, clearly demonstrating how outcomes in turn influence causes. The following sections will elaborate on these six feedback loops in detail.

#### 2.2.1 Digital empowerment of health feedback loop

**Figure 2:**
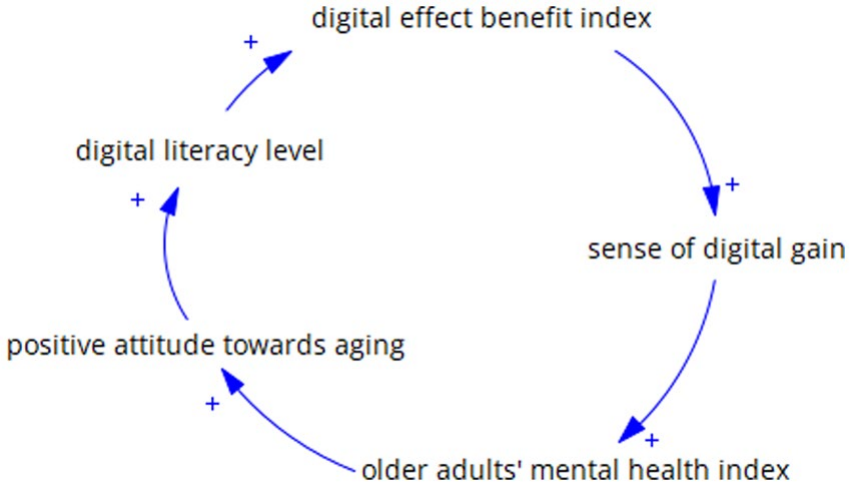
Digital empowerment of health feedback loop.

Digital empowerment of health feedback loop aligns with the theory of self-efficacy in public health (45), as shown in Figure 2. The improvement in digital literacy among older adults enables better use of digital devices, thereby increasing the depth of digital usage. This increased depth of digital usage promotes a rise in the digital effect benefit index for older adults, subsequently enhancing their sense of digital gain. The heightened sense of digital gain boosts the satisfaction of older adults, thereby improving their mental health and increasing their mental health index. An improved mental health index helps reduce anxiety caused by the digital divide, encouraging a positive attitude toward digital technology and increasing the positivity of aging attitudes (46). The enhancement in the positive attitude toward aging further promotes the improvement of digital literacy, thereby forming a positive feedback loop that continuously enhances self-efficacy.

#### 2.2.2 Health promotion digital engagement feedback loop

**Figure 3:**
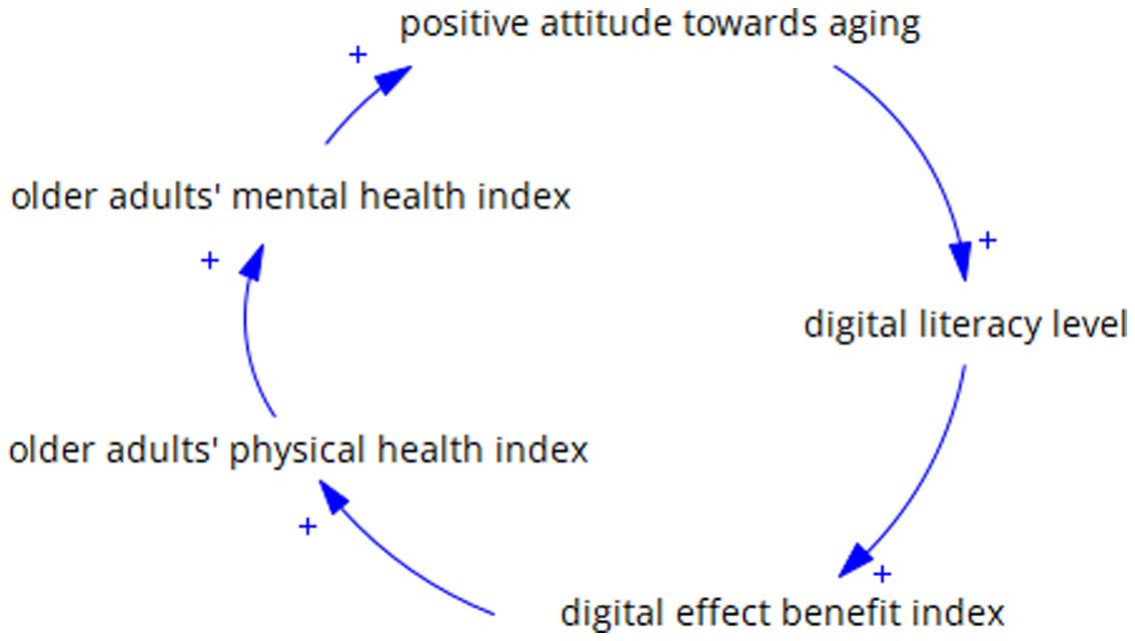
Health promotion digital engagement feedback loop.

The digital engagement feedback loop for health promotion aligns with the health life course theory in the public health field, demonstrating the feedback among health levels, cognitive attitudes, and health behaviors (47), as shown in Figure 3. The mental health the older adults influences their perceptions of aging, with those having higher mental health indices showing more positive attitudes toward aging (48). Increased positive attitudes toward aging encourage the older adults to adopt an inclusive attitude toward digital technology, motivating them to learn and absorb digital skills more actively to enhance their digital literacy. Boosted digital literacy empowers senior citizens to obtain medical advice more efficiently via electronic tools, promoting superior physical health. Improved physical health indices, subsequently, assist in elevating the mental health indices of older adults (49).

#### 2.2.3 Social support reinforcement feedback loop

**Figure 4:**
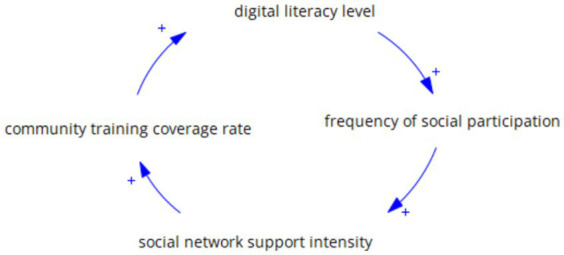
Social support reinforcement feedback loop.

The reinforcement feedback loop of social support aligns with social capital theory in the public health domain, involving both structural and cognitive social capital (50), as shown in Figure 4. Can be dispelled by social support through the older adults citizens’ adaptation to the digital atmosphere, increasing the point coverage rates of the community training, and thus improving the older adults citizens’ digital skills and increasing the frequencies of participation in social activities (51). The more often older adults participate socially, the stronger their social network support intensity, and when they have problems that arise they will be helped in a timely manner. A more involved group of older adults will heighten community attention and concern for this group and funding for community training will increase. Consequently, the coverage of community training expands (52).

#### 2.2.4 Online risk mitigation feedback loop

**Figure 5:**
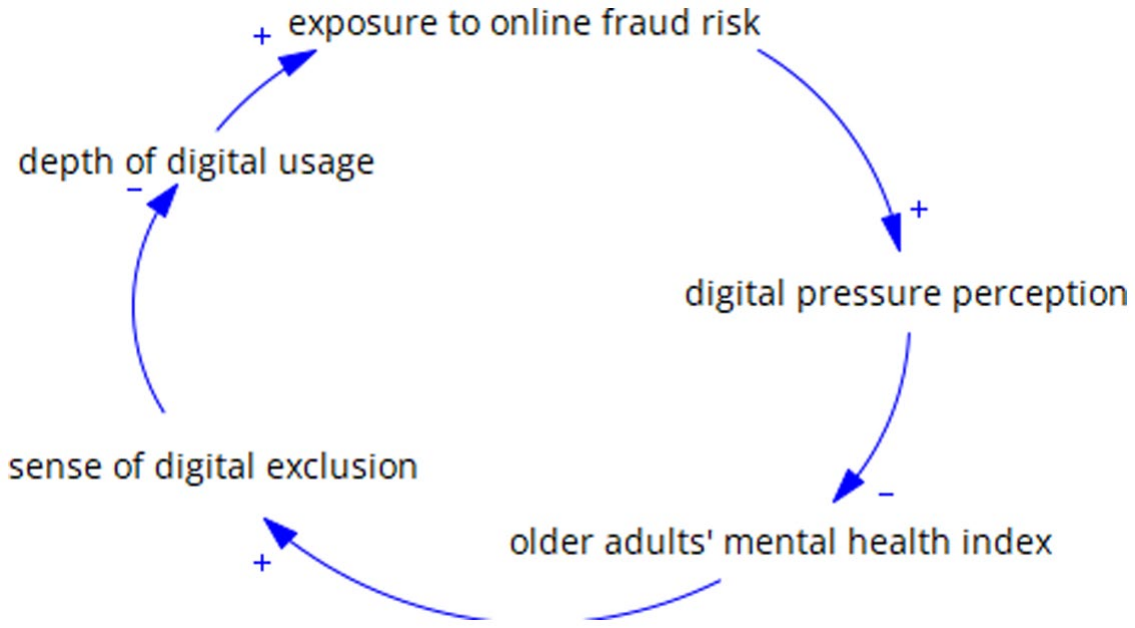
Online risk mitigation feedback loop.

The online risk mitigation feedback loop is an adaptive mechanism that prevents the manifestation of psychological resilience by reducing the use of digital technology among older adults with limited psychological resilience, aligning with psychological resilience theories in public health (53), as shown in Figure 5. As older adults increase their depth of digital usage, they are exposed to more information, which creates opportunities for malicious actors to infiltrate, thereby increasing their exposure to online fraud risks. The heightened exposure to online fraud risks can lead to feelings of anxiety and panic when using digital technologies, increasing older adults’ perception of digital stress, which in turn lowers their mental health index (54). The decline in older adults’ mental health index leads them to distance themselves from the digital environment to reduce online fraud risks, thereby increasing their sense of digital exclusion. The increased sense of digital exclusion reduces their depth of digital usage, exacerbating the digital divide among older adults.

#### 2.2.5 Information narrowing exclusion feedback loop

**Figure 6:**
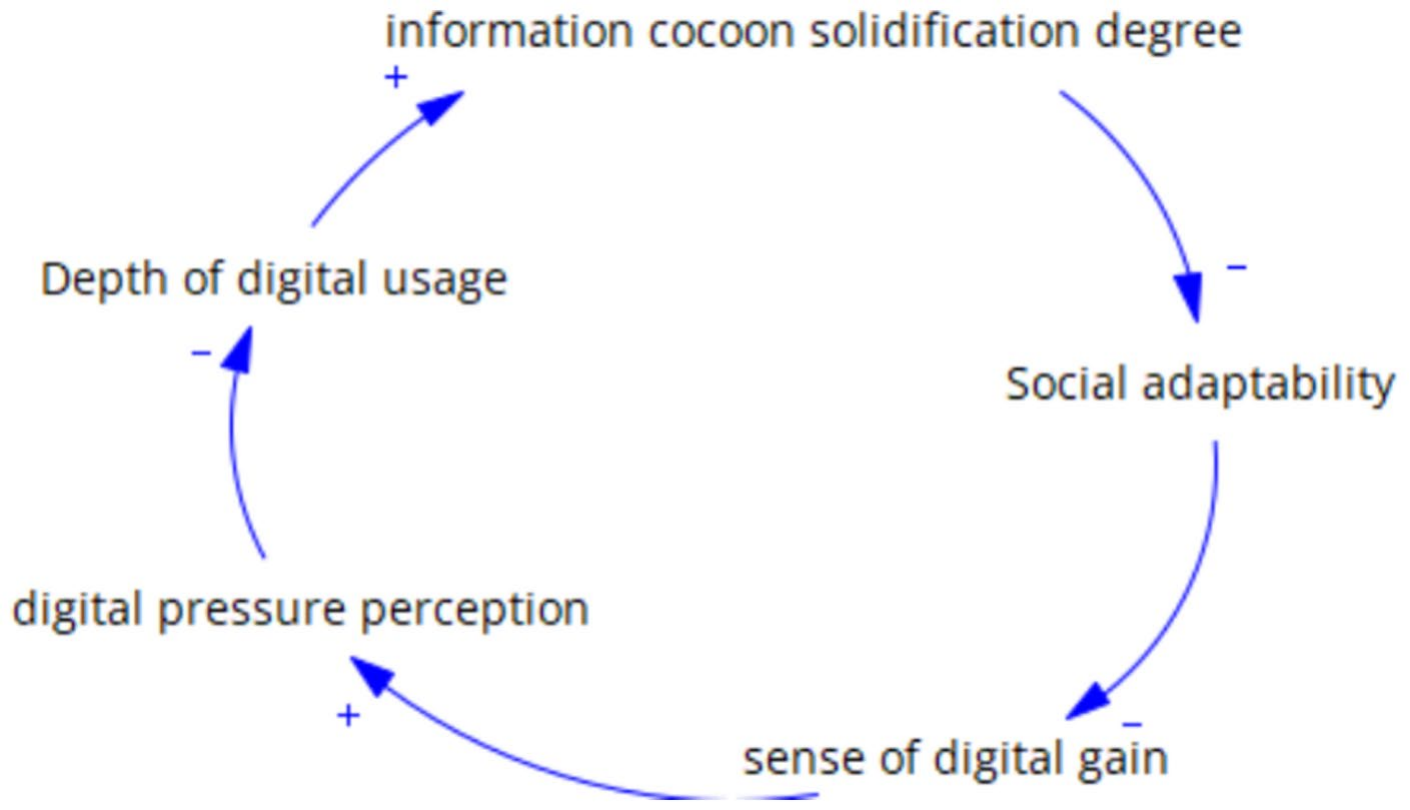
Information narrowing exclusion feedback loop.

The information narrowing exclusion feedback loop aligns with the Health Belief Model in the field of public health, encompassing elements such as perceived threats in the HBM (55), as shown in Figure 6. Information cocoons arise when users selectively accept information, resulting in boundaries being put on the scope of information acquisition and utilization (56). As the digital usage deepens, thus social networks tend to recommend like types of information, causing this population to spend longer in fairly homogeneous information environments increasing the rigidity of information cocoons, so the social adaptability of which reduces, and their sense of digital gain declines, and they are less willing to tap digital tools, thus has led to a dull down of a digital usage depth.

#### 2.2.6 Digital dependency loss feedback loop

**Figure 7:**
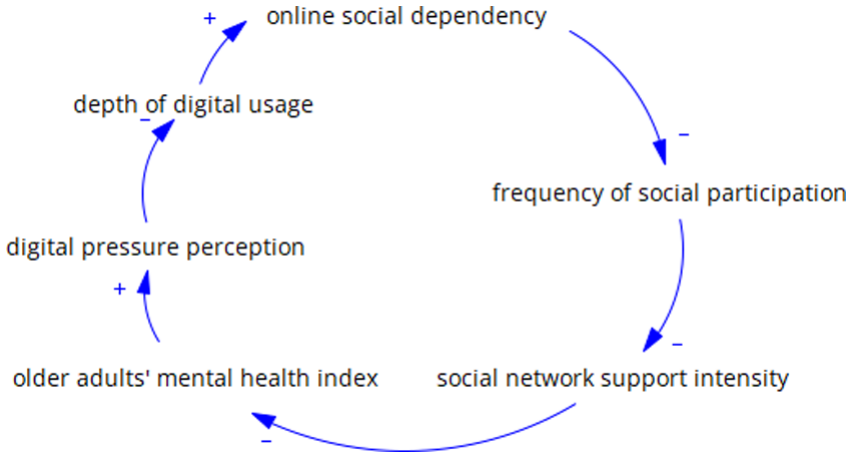
Digital dependency depletion feedback loop.

The digital dependency depletion feedback loop corresponds to the Conservation of Resources theory in public health, indicating that excessive investment in one resource leads to the loss of other resources, resulting in stress and behavioral regression decline (57, 58), as shown in Figure 7. Increased depth of digital usage leads to a rise online social dependence among older adults, reducing face-to-face communication in real life, decreasing the frequency of social participation, and weakening the strength of social network support. This makes older adults more prone to loneliness and lowers their mental health. The decline in mental health index, in turn, increases digital pressure perception, further driving a decrease in the depth of digital usage, forming a negative feedback loop (59, 60).

### 2.3 Research methodology

System dynamics involves constructing stock-flow diagrams, establishing variable simulation equations, and simulating the complex dynamic evolution processes of variables (61). It is particularly suitable for theory-building research, especially in simulation problems where data is insufficient (62–64). This study constructs four subsystems, namely the digital divide, physical and mental health, mediating mechanisms, and risk regulation. These subsystems interact with each other, resulting in nonlinear dynamic relationships among the variables. Additionally, the mechanism by which the digital divide affects the physical and mental health of older adults is a dynamic process, and system dynamics excels at simulating long-term dynamic impact processes. Therefore, system dynamics can effectively simulate this system, making this study feasible.

### 2.4 Model construction

#### 2.4.1 Development of causal loop diagrams and expert involvement

This study first adopts an integrated theoretical framework, summarizing core mechanisms such as digital empowerment, social support, and network risks into six feedback loops. Based on this, an initial causal loop diagram is drawn, clarifying the polarity relationships and feedback closure paths among variables. On this basis, the Delphi method is employed to conduct expert validation of the model boundaries and loop settings, inviting five experts from the fields of the digital divide, aging health, and public health policy to participate in two rounds of back-to-back reviews. In the first round of consultation, the experts’ recognition rate for the completeness of the model boundaries and the rationality of the loop structure was 85%. Based on feedback, the “information narrowing exclusion feedback loop” was added, and the variable relationships in the “online risk mitigation feedback loop” were refined. In the second round of review, the experts’ agreement rate on the overall composition of the revised loops reached 100%, thereby determining the final causal loop structure. A systematic comparison of the theoretical basis, critical pathways, loop polarity, and main functional stages of the six feedback loops is shown in Table 2.

**Table 2:** Comparison of feedback loops in the digital divide’s impact on the physical and mental health of older adults.

Feedback loop	Theoretical foundation	Circuit polarity	Main action phase
Digital empowerment of health feedback loop	self-efficacy	Positive	Initial (0–20)
Health promotion digital engagement feedback loop	Healthy life course theory	Positive	The whole period
Social support reinforcement feedback loop	Social capital theory	Positive	Middle to late stage
Online risk mitigation feedback loop	Psychological resilience theory	Negative	Medium-term (20–70)
Information narrowing exclusion feedback loop	Health belief model	Negative	Medium-term (20–70)
Digital dependency depletion feedback loop	Conservation of resources theory	Negative	Later period

#### 2.4.2 Construction of stock-flow diagrams

**Figure 8:**
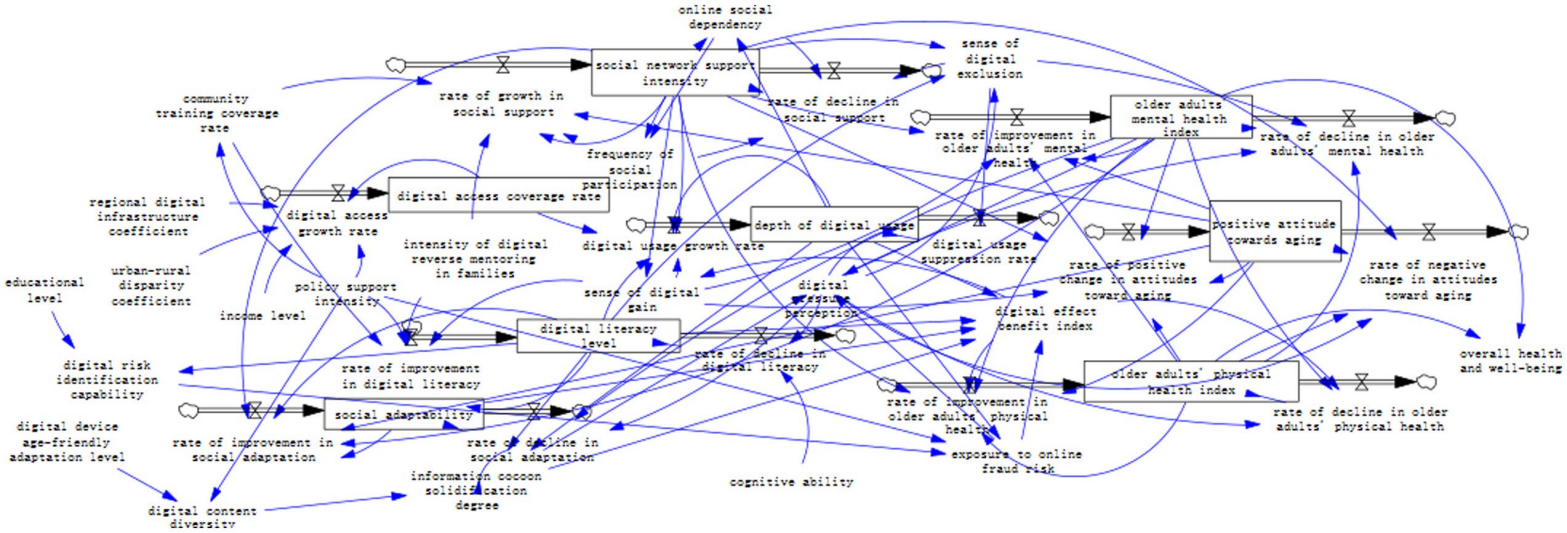
Stock and flow diagram of the system dynamics model.

Based on the causal loop diagram and following system dynamics modeling conventions, the digital divide, physical and mental health, mediating mechanisms, and risk regulation were divided into four subsystems. Key concepts in the loops were converted into quantifiable stocks, flows, and auxiliary variables, and a stock–flow diagram was drawn using Vensim PLE. The model includes 8 stocks, 15 flows, 12 auxiliary variables, and 8 constants, with dynamic coupling among variables implemented via simulation equations, as shown in Figure 8.

### 2.5 Main parameters and simulation equations of the model

This section presents the main parameters and simulation equations of the model according to the system configuration.

#### 2.5.1 Main parameters

This model includes a total of 43 variables. The variable names, their properties, and the initial values of related variables are shown in Table 3.

**Table 3:** Model parameters and their properties.

Variable name	Property	Initial value
Digital literacy level	Stock	0.35
Digital access coverage rate	Stock	0.52
Depth of digital usage	Stock	0.2
older adults’ physical health index	Stock	0.5
Older adults’ mental health index	Stock	0.5
Positive attitude toward aging	Stock	0.5
Social adaptability	Stock	0.5
Social network support intensity	Stock	0.5
Rate of improvement in digital literacy	Flow	-
Rate of decline in digital literacy	Flow	-
Digital access growth rate	Flow	-
Digital usage growth rate	Flow	-
Digital usage suppression rate	Flow	-
Rate of improvement in Older adults’ physical health	Flow	-
Rate of decline in Older adults’ physical health	Flow	-
Rate of improvement in Older adults’ mental health	Flow	-
Rate of decline in Older adults’ mental health	Flow	-
Rate of positive change in attitudes toward aging	Flow	-
Rate of negative change in attitudes toward aging	Flow	-
Rate of improvement in social adaptation	Flow	-
Rate of decline in social adaptation	Flow	-
Rate of growth in social support	Flow	-
Rate of decline in social support	Flow	-
Policy support intensity	Constant	0.5
Urban–rural disparity coefficient	Constant	0.8
Regional digital infrastructure coefficient	Constant	0.8
Educational level	Constant	0.5
Income level	Constant	0.5
Digital device age-friendly adaptation level	Constant	0.45
Intensity of digital reverse mentoring in families	Constant	0.4
Cognitive ability	Constant	0.8
Digital risk identification capability	Auxiliary	–
Exposure to online fraud risk	Auxiliary	–
Digital content diversity	Auxiliary	–
Information cocoon solidification degree	Auxiliary	–
Online social dependency	Auxiliary	–
Frequency of social participation	Auxiliary	–
Digital pressure perception	Auxiliary	–
Digital effect benefit index	Auxiliary	–
Sense of digital gain	Auxiliary	–
Sense of digital exclusion	Auxiliary	–
Community training coverage rate	Auxiliary	–
Overall health and well-being	Auxiliary	–

#### 2.5.2 Simulation equations

The simulation equation coefficients for the variables discussed in this paper are determined at three levels. First, if existing empirical studies directly report standardized path coefficients or effect sizes, these are adopted directly and normalized. Second, if no direct effect size is available, initial values are estimated based on the variable’s explanatory power relative to other variables within the relevant theoretical framework, using an equal-weight allocation principle. Third, the decay rate and inhibition rate coefficients are set based on the “annual loss rate of low-level variables” reported in the literature, adjusted for time scale. All coefficients undergo robustness testing during the model calibration phase by repeatedly adjusting them by ±10%. When the output variable is insensitive to a coefficient change, the baseline value is retained; if sensitive, the coefficient’s range is further refined through literature review. Therefore, the simulation equation formulas and their basis are shown in Table 4.

**Table 4:** Simulation equation formulas for variables.

Variable name	Simulation equation formula	Programming basis
Digital literacy level	INTEG (Rate of improvement in digital literacy-rate of decline in digital literacy)	Initial value 0.35---older adults56th Statistical Report on Internet Development in China.
Digital access coverage rate	INTEG (Digital access growth rate)	Initial value 0.5---The 56th Statistical Report on Internet Development in China.
Depth of digital usage	INTEG (Digital usage growth rate-digital usage suppression rate)	Initial value 0.2--- older adultsRE]older adults’[/iC : RE] lower initial depth of use, based on DiMaggio & Hargittai discussion of the “quality of use gap” within the secondary digital divide, synthesizes multiple survey data on online time, number of applications, and operational complexity among older adults population (12).
Older adults’ physical health index	INTEG (rate of improvement in Older adults’ physical health-rate of decline in Older adults’ physical health)	Initial value 0.5---the mean self-rated health score of older adults in the 2018 national sample of the China Health and Retirement Longitudinal Study (CHARLS), representing a moderate level of physical functioning.
Older adults’ mental health index	INTEG (rate of improvement in Older adults’ mental health-rate of decline in Older adults’ mental health)	Initial value 0.5---based on the composite mean of the Geriatric Depression Scale (GDS) and Positive Affect Scale scores, combined with data from the CHARLS mental health module, the central level is set at 0.5.
Positive attitude toward aging	INTEG (rate of positive change in attitudes toward aging-rate of negative change in attitudes toward aging)	Initial value 0.5---based on the median of the Chinese norm of the Attitudes to Aging Questionnaire (AAQ) and the average positive aging attitude scores of older adults in relevant empirical studies.
Social adaptability	INTEG (rate of improvement in social adaptation-rate of decline in social adaptation)	Initial value 0.5---moderate adaptation level, set based on the mean social adaptation assessment of community-dwelling older adults.
Social network support intensity	INTEG (rate of growth in social support-rate of decline in social support)	Initial value 0.5---based on the Lubben Social Network Scale-6 score, combined with the average level of social support among older adults in Chinese urban communities.
Rate of improvement in digital literacy	(intensity of digital reverse mentoring in families*0.3 + community training coverage rate*0.3 + policy support intensity*0.2 + sense of digital gain*0.2) *(1-digital literacy level) *0.1	Family, community training, policies, and sense of digital gain promote the enhancement of digital literacy.
Rate of decline in digital literacy	[(1-cognitive ability) *0.02 + digital pressure perception*0.02] *digital literacy level	Cognitive ability and digital pressure affect it.
Digital access growth rate	(policy support intensity*0.3 + income level*0.2 + "urban–rural disparity coefficient”*0.2 + regional digital infrastructure coefficient*0.3) *(1-digital access coverage rate) *0.2	Kumar et al. found that infrastructure and policy are the primary determinants of digital access (67). Income levels and urban–rural disparities are secondary economic and social factors (21, 68).
Digital usage growth rate	(digital literacy level*0.3 + digital access coverage rate*0.3 + social network support intensity*0.2 + sense of digital gain*0.2) *(1-depth of digital usage) *0.15	Digital access and digital literacy are the material prerequisites and competency foundations for digital usage, respectively (69). The strength of social network support facilitates technology use among older adults (70). Wang, using an SEM–ANN empirical analysis, found that perceived usefulness has a positive effect on digital usage (71).
Digital usage suppression rate	(sense of digital exclusion*0.05 + digital pressure perception*0.05) *depth of digital usage	Digital exclusion significantly negatively predicts older adults’ willingness to continue using digital technology. Empirical research by Cheshmehzangi et al. indicates that perceived digital stress is the main reason for older adults withdrawing from the digital environment (54).
Rate of improvement in older adults’ physical health	(digital effect benefit index*0.3 + older adults’ mental health index*0.2 + social network support intensity*0.2 + positive attitude toward aging*0.1)*(1-older adults’ physical health index)*0.15	The National Health Commission’s “The standard for healthy Chinese older adults” clearly states that physical health is most significantly influenced by the digital effect benefit index. Social network support has a direct protective effect on the physical health of the older adults’ (72). Based on 8 years of HRS data, a bidirectional mutual promotion mechanism between physical and mental health was identified (73). The positive impact of aging attitudes on physical health is relatively indirect (74).
Rate of decline in older adults’ physical health	((1-digital effect benefit index) *0.02 + digital pressure perception*0.02 + (1-older adults’ mental health index) *0.02) *older adults’ physical health index	”The standard for healthy Chinese older adults” issued by the National Health Commission specifies that health assessment includes physical health, mental health, and social health, with the three functionally influencing each other.
Rateolder adults’of improvement in older adults’ mental health	(sense of digital gain*0.3 + social network support intensity*0.3 + positive attitude toward aging*0.2 + older adults physical health index*0.2) *(1-older adults’ mental health index) *0.15	The direct promotion effect of digital sense of gain and social network support on mental health; Park et al. found that the standardized path coefficient of social network support on older adults’ mental health is approximately 0.3 (75). Aging attitudes and physical health are relatively indirect factors in improving the audit health of the older adults (76).
Rate of decline in older adults’ mental health	(digital pressure perception*0.1 + sense of digital exclusion*0.05 + (1-older adults’ physical health index) *0.02) *older adults’ mental health index	The “digital risk-psychological stress-health damage” pathway model by Cheshmehzangi et al. confirms that perceived digital stress is the primary modifiable factor in the deterioration of older adults’ mental health (54).
Rate of positive change in attitudes toward aging	(older adults’ mental health index*0.3 + social network support intensity*0.2 + sense of digital gain*0.2 + older adults’ physical health index*0.1) *(1-positive attitude toward aging) *0.1	Sabatini et al., based on 20-year longitudinal data from the German ILSE study, found that mental health is a core variable in older adults’ attitudes toward aging (77). Social network support and digital sense of gain indirectly shape positive aging attitudes by enhancing life satisfaction (78, 79).
Rate of negative change in attitudes toward aging	(digital pressure perception*0.1 + (1-social network support intensity) *0.05 + (1-older adults physical health index) *0.02) *positive attitude toward aging	Digital pressure perception significantly enhances negative aging attitudes among older adults’ (80).
Rate of improvement in social adaptation	(social network support intensity*0.3 + older adults’ mental health index*0.2 + digital literacy level*0.2 + positive attitude toward aging*0.2) *(1-social adaptability) *0.1	Social network support is the foundation for building social adaptability in older adults (81). Digital literacy levels and mental health help older adults better adapt to society (82, 83).
Rate of decline in social adaptation	[digital pressure perception*0.05 + (1-older adults’ mental health index) *0.05 + information cocoon solidification degree*0.05] *social adaptability	Information narrowing can limit an individual’s cognitive perspective and social adaptability, and when combined with digital pressure and psychological vulnerability, it collectively erodes social adaptive capacity (84).
Rate of growth in social support	(frequency of social participation*0.3 + intensity of digital reverse mentoring in families*0.3 + community training coverage rate*0.2 + positive attitude toward aging*0.1) *(1-social network support intensity) *0.1	Greenfield and Marks found that the frequency of social participation is the primary predictor of social support for older adults in the community (52). Chung et al. confirmed that family digital feedback ability directly strengthens older adults sense of being supported (85).
Rate of decline in social support	[(1-frequency of social participation) *0.05 + sense of digital exclusion*0.05 + online social dependency*0.02] *social network support intensity	Online social dependence and frequency of social participation are consistent with the findings of Lieberman & Schroeder that “online substitution for offline leads to weakened support (86).”
Digital risk identification capability	educational level*0.3 + digital literacy level*0.7	Shang et al., in a review based on protection motivation theory, found that digital literacy level is a core predictor of risk perception and prevention ability among the older adults with a relative weight approximately 2.3 times that of education level (6).
exposure to online Fraud risk	(depth of digital usage*0.5 + (1-digital risk identification capability) *0.5) *(1-policy support intensity*0.3)	Shang et al. pointed out that online fraud victimization is the product of the combined effects of “exposure opportunity” and “defensive capability,” with both factors having equal influence (6). Therefore, the depth of digital use and the lack of digital risk identification ability are both 0.5.
Digital content diversity	policy support intensity*0.3 + "digital device age-friendly adaptation level”*0.5 + 0.2	0.2 is the baseline diversity, and age-friendly products and related policies will increase digital content diversity.
Information cocoon solidification degree	depth of digital usage*0.4*(1-digital literacy level*0.5) *(1-digital content diversity)	Digital content diversity and the level of digital literacy can reduce the solidification of information cocoons, while the depth of digital usage can exacerbate it.
Online social dependency	depth of digital usage*0.6 + (1-social network support intensity) *0.4	Digital usage may replace offline social interactions and increase online social dependency, while stronger social network support can reduce online social dependency.
Frequency of social participation	social network support intensity*0.5 + (1-online social dependency) *0.5	Social network support intensity promotes social participation, while the degree of online social dependence reduces social participation.
Digital pressure perception	exposure to online fraud risk*0.4 + information cocoon solidification degree*0.3 + (1-older adults’ mental health index) *0.3	The study by Cheshmehzangi et al. indicates that cybersecurity risks are the primary external source triggering digital stress among older adults (54); information cocoons exacerbate stress through cognitive narrowing.
Digital effect benefit index	MAX (0, digital literacy level*0.4 + depth of digital usage*0.3 + social adaptability*0.3-information cocoon solidification degree*0.2-exposure to online fraud risk*0.1)	According to Van Deursen and Helsper’s three-level digital divide theory, the outcome gap primarily depends on users’ digital literacy levels, followed by the depth of digital usage (13). Social adaptability serves as an important mediator in converting digital benefits (87).
Sense of digital gain	MAX (0, digital effect benefit index*0.6 + social network support intensity*0.4)	Sense of digital gain is digital effect benefit and social network support, with digital effect benefits being predominant.
Sense of digital exclusion	(1-digital literacy level) *0.3 + digital pressure perception*0.4 + (1-social network support intensity) *0.3	Low digital literacy, high digital pressure, and low social network support constitute a sense of digital exclusion (88).
Community training coverage rate	policy support intensity*0.6 + 0.2	0.2 is the baseline coverage rate, and policy support influences the community training coverage rate.
Overall health and well-being	older adults’ mental health index*0.5 + older adults’ physical health index*0.5	According to the WHO definition of health, health is a state of complete physical, mental, and social well-being. In this model, social adaptability indirectly affects overall health and well-being by influencing physical and mental health, so overall health and well-being is divided into two dimensions: physical and mental (89). Haskell et al. also adopted an equal weighting method when constructing a comprehensive health indicator (76).

### 2.6 Simulation setup and model configuration

This study completed model construction and simulation runs in the Vensim PLE environment. The simulation time range is 100 months, with a simulation step size set to 0.25 months. The integration method employs the fourth-order Runge–Kutta method to ensure solution accuracy for the nonlinear feedback system.

The model baseline simulation operates under the following assumptions:

(1) External intervention variables such as policy support intensity, family digital feedback intensity, and the degree of age-friendly digital devices remain at their initial constant values throughout the simulation period to observe the system’s endogenous dynamics.
(2) The initial values of each stock variable are calibrated based on literature and statistical data, assuming that the system has not yet experienced large-scale external shocks at the start of the simulation.
(3) The causal weights between variables are set according to the direction and relative magnitude of effects reported in existing empirical studies, and are calibrated within a reasonable range, without introducing time-varying coefficients.

Sensitivity analysis run settings: Based on the baseline simulation, single-factor sensitivity tests are conducted on three parameters: policy support intensity, family support strength, and the age-friendliness of digital devices. Each time, only one target parameter is adjusted, increasing or decreasing its value by 60% relative to the initial value, while all other parameters and simulation settings remain consistent with the baseline run. By comparing the evolution paths of overall health and well-being under different scenarios, the most sensitive key variables are identified.

## 3 Results

### 3.1 Model validation

To ensure that the simulation model effectively reflects the real-world system, this study validates it from two aspects: structural validity and behavioral validity.

In terms of structural validity, both the model structure and equations are supported by established theories, and the direction of relationships and the range of relative weights among variables are set based on existing empirical literature. On this basis, extreme condition tests are conducted by setting key variables, such as policy support intensity and digital literacy level, to extreme values of 0 and 1, to examine whether the model produces boundary behaviors consistent with theoretical expectations. The results show that under extreme conditions, the model does not generate meaningless numerical overflow or logical paradoxes.

In terms of behavioral validity, the overall health and well-being evolution trend simulated by the model is qualitatively compared with the approximate time-series trend of self-rated health among older adults reported in the China Blue Book on Older Adult Health and the CHARLS survey. The simulation results indicate that overall health and well-being improves rapidly in the early stage, slows down in the mid-to-late stage, and gradually stabilizes. This pattern is generally consistent with the actual data trend observed in China between 2020 and 2025, where the self-rated health rate among older adults increased year by year but with a narrowing marginal increase. The value ranges of key variables also align with those reported in existing empirical studies, without systematic deviation. The model can reproduce the historical behavioral patterns of the real-world system with reasonable accuracy and is capable of conducting policy simulations and dynamic projections.

### 3.2 Model result analysis

**Figure 9:**
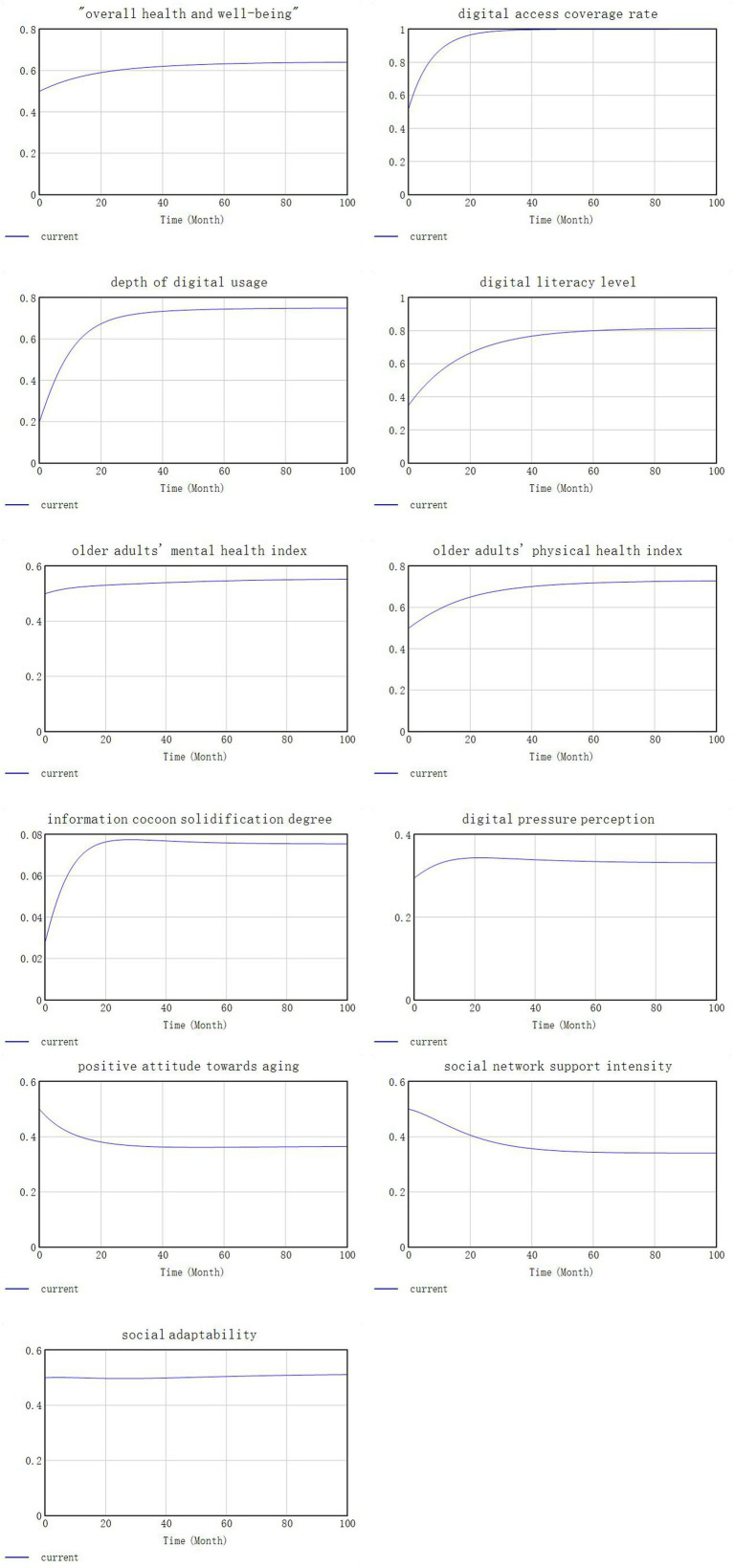
Basic results of model operation.

Input the above parameters and equations into the system model, set the simulation period to 100 months, and the basic operational results of the simulation are shown in Figure 9.

From the simulation results, the initial phase (0–20) is a period where digital technology continuously integrates into the lives of older adults while digital risks emerge. Empowerment for health is the predominant model, with improvements in proxy indicators such as digital access coverage rate, digital literacy, and depth of digital usage. As healthy old people become more steeped in the digital environment, they become more vulnerable to the negative effects of the digital world, experiencing digital risks, leading to increases in perceived digital pressure, and cementing information cocoons. This exacerbates the decline in a positive attitude toward aging, but the power of empowerment at this stage is through level of digital literacy for improving health for old people. The burden of digital technology is lower than the benefits, and this improves the indices of mental health and physical health, improving health overall.

In the mid-phase (20–70), the Online risk mitigation feedback loop and the Information narrowing exclusion feedback loop will gradually plays a stabilizing role and create a balance, that is, a stasis occurs between the positive feedback loop and the negative feedback loop. Overall health and well-being increase from 0.59 to 0.64, but the rate of increase gradually slows. The reason for this is that the coupling of variables is still going on during dynamic change, and the system will gradually stabilize. Digital pressure perception drops from 0.34 to about 0.33 and stabilizes. In short, the negative impact still exists, but it is continuously mitigated by positive feedback. Information cocoon solidification degree peaks at about the 28th month and begins to slow down. This indicates that older adults experience an aggravation of information cocoons at the beginning due to repeated information, but they are aware after the digital literacy improves and try to get rid of the information cocoon by digital content diversification. Social network support intensity drops to 0.34 and stabilizes. This reflects that older adults use digital technology more extensively, and their online social dependency increases, which lowers social network support intensity. Over time, older adults eventually come to a balance between the two.

In the later phase (70–100), older adults have become relatively familiar with the digital environment, and various variables tend to stabilize. The older adults’ physical health index is approximately 0.73, and the mental health index is around 0.55, with both showing an increase of less than 0.01. This keeps the overall health and well-being maintained at around 0.64. Simultaneously, a discrepancy persists near 0.2 regarding the physical health index and the mental health index for older adults, suggesting that within the investigation of how the digital divide affects the physical and mental health of older adults, physical health dominates over mental health.

### 3.3 Sensitivity analysis

**Figure 10:**
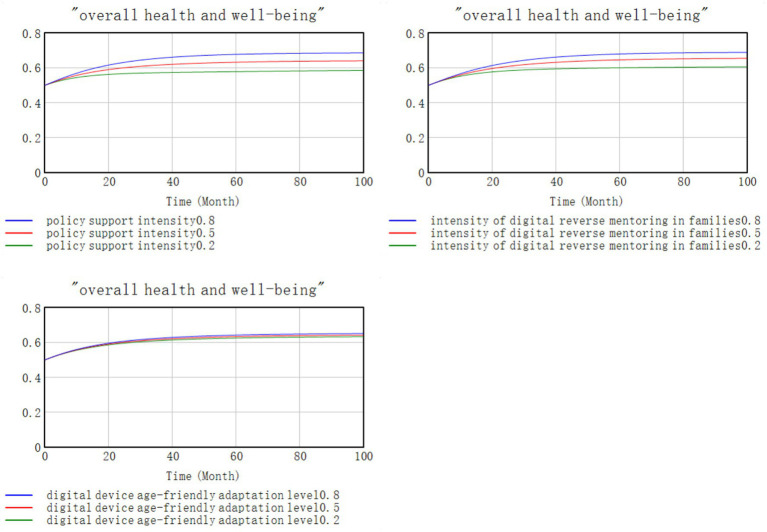
Policy, family, digital device age-friendly sensitivity analysis.

Sensitivity analysis refers to changing the parameter of only one variable while keeping all other variables constant, in order to observe the dynamic changes of other variables during the model’s operation. It is particularly suitable for application in simulation models (65, 66). In the system dynamics model of this study, the main factors affecting the overall health and well-being of older adults are policy support intensity, the intensity of digital reverse mentoring in families, and the digital device age-friendly adaptation level for older adults. Therefore, sensitivity analysis is conducted by separately changing the parameters of these three variables to identify the sensitive factors influencing overall health and well-being. The simulation results are shown in Figure 10.

Simulation results indicate that, in terms of policy support intensity, an increase in such intensity leads to an improvement in community training coverage rate and a reduction digital pressure perception, thereby positively influencing overall health and well-being. As the intensity of digital reverse mentoring in families, it accelerates the growth rate of digital literacy and social network support intensity, thereby contributing to an increase in overall health and well-being. As for the digital device age-friendly adaptation level, a high digital device age-friendly adaptation level to content diversity, which in turn improves overall health and well-being. Overall, the simulation verification results show that policy support intensity is the most sensitive factor, followed by the intensity of digital reverse mentoring in families, while the digital device age-friendly adaptation level exhibits weaker sensitivity. Therefore, policy support intensity is the key factor in addressing the digital divide among older adults and enhancing their physical and mental health.

## 4 Discussion

### 4.1 Main conclusion

This research utilizes the system dynamics technique to simulate and perform sensitivity evaluations on the constructed system dynamics model examining how digital divide impact the physical and mental health of older adults, resulting in the subsequent primary findings.

First, in the study of the mechanisms by which the digital divide affects the physical and mental health of older adults physical health takes precedence over mental health, with the older adults’ physical health index being approximately 0.2 higher than their mental health index. This is because physical health is examined through objective lab-tabulation and mental health is perceived through subjective feelings. Thus, digital technologies have a faster effect on the physical health than mental health where cultivation need the long-term contribution of social systems. Since the two types of health are thus affected by different primary means, the physical health of older adults will tend to close the digital divide faster than their mental health. To narrow the gap, it is important to put attention to the current lack of digital mental health services, to create digital mental health platforms for older adults and cooperate with relevant departments to create age-appropriate mental health apps, which should be important aspects of future intervention policies.

Furthermore, creating a more harmonious equilibrium between online and offline social engagement can foster the physical and mental health of older adults. Virtual social networking bypasses spatial limitations for communal interaction and connects the aged with relatives who reside outside their own homes. However, high use intensity increases the risk of online social interaction being too much of a good thing for older adults, leading to lower frequency of social participation, less social network support and lower mental health index significantly. Therefore, while bridging the digital divide, it is also important to emphasize offline social interactions, preventing digital technology from completely disrupting traditional social relationships and achieving a balance between online and offline social engagement.

Third, narrowing the digital divide exerts a general beneficial influence on the physical and mental health of older adults, where digital literacy acts as a pivotal catalyst for enhancing their quality of life. Concurrently, the effect of this digital divide on the physical and mental health of older adults is dual-sided. However, these risks can be effectively managed.

Fourth, policy support intensity is most sensitive to overall health and well-being, serving as a critical factor in bridging the digital divide and promoting the physical and mental health of older adults. In the future, if we are to stop the digital divide becoming a wider gulf, and to improve the physical and mental health of older adults, we will need to invest more policy support in initiatives like “embedding digital technologies in community public service packages” and “creating safety nets in communities to protect older people from online fraud”.

### 4.2 Theoretical contribution

Existing academic research lacks comprehensive exploration into the influence of the digital divide on the physical and mental health of older adults, with most existing works remaining static instead of dynamic. Drawing on the system dynamics approach, this article develops a system dynamics model illustrating how this digital divide affects older adults’ health status. It employs system dynamics techniques to measure correlations between factors like digital literacy level, depth of digital usage, social network support intensity, and social adaptability, modeling the developmental trajectory of how this digital divide influences the health mechanisms of older adults. This investigation provides multiple theoretical insights.

First, it opened the “black box” by dynamically simulating the pathway from digital literacy to overall health and well-being through two mediating variables, namely positive attitude toward aging and social network support. This illustrated how digital components and their intermediary factors influence overall health and well-being of life, enhancing the conceptual basis for studies concerning the mechanisms of digital empowerment for older adults individuals’ overall health and well-being, while expanding and intensifying the breadth of inquiry regarding how the digital divide impacts the physical and mental health of older adults.

Furthermore, it was observed that enhancing digital literacy exerts a beneficial impact upon the physical and mental health of older adults. This finding offers a substantial conceptual advancement for the disciplines of digital health and dynamic management. The investigation into the process through which the digital divide impacts the physical and mental health of older adults departs from the stagnant viewpoints of prior inquiries. By utilizing the framework of system dynamics, it overcomes the constraints in temporal analysis, illustrates the trends of how the digital divide affects the progression of the physical and mental health mechanisms of older adults, and further enriches both the capacity theory and dynamic theory.

Third, it enriches the application of system dynamics in the field of digital health. This study reveals that digital technology serves as both a core driver for improving the physical and mental health of older adults and a source of digital risks, significantly increasing the risks of online fraud and information cocoons, thereby demonstrating its dynamic balancing mechanism.

### 4.3 Practical significance

This study focuses on the older adult population and constructs a system dynamics model to provide practical guidance on how the digital divide affects their physical and mental health, with the following two practical implications. First, rational allocation of limited resources: according to the system dynamics model, the level of policy support has the greatest impact on the overall health and well-being of older adults Therefore, the government should strengthen policy support to ensure resources are effectively implemented across all aspects of society, build a digital inclusion system, accelerate the construction of community digital infrastructure, integrate digital technology training into community public health services, and establish digital technology instructors to regularly assist older adults with online appointment registration and the use of health-related apps, thereby enhancing their digital literacy and physical and mental health. At the same time, community property management, neighborhood police stations, banks, and other entities should be encouraged to form anti-fraud publicity teams, regularly visit communities to educate older adults about typical online fraud cases, promote awareness of online fraud prevention, reduce digital risk behaviors, and thus improve the physical and mental health of older adults Moreover, you should not rely too much on online social dependency. Life online will not replace life offline. Life as usual should be a combination of online and offline. You have to combine digital technology in existing social events. Community have to have events that need to sign up online and come to join offline. For instance, posting health lectures in WeChat group, guide older adults learning online through IT appliances. And develop the online and offline combination. They will not lose the frequency of offline social participation. They can keep their social participation levels and digital literacy high.

### 4.4 Limitations and future directions

Although methodological advancements were made, this research possesses four specific constraints. Initially, because of the challenges in quantifying factors, the parameter configurations for the system dynamics model constructed here to investigate how the digital divide impacts the physical and mental health of older adults remain rather basic. Differences among older adults in terms of education level and cognitive ability were not considered and were simply treated as constants. This does not reflect the differences in digital inclusion and risk judgment ability ofolder adults with different educational backgrounds, nor the changes in older adults population with age. A future study may break through the quantitative bottleneck of system dynamics models by investigating the different performances of older adults’ groups with different education levels in such models, and incorporate age as the variable to study the mechanism of the digital divide affecting the physical and mental health of older adults, while studying the influence of age on cognitive ability and the effect of cognitive ability on digital literacy. Secondly, this study did not consider external factors, such as the occurrence of public emergencies. Public health emergencies may affect the scope of digital technology usage over a given period for a specific region, future studies could use the public emergency intensity variable to approximately simulate these effects on the variables such as the depth of digital technology usage, and health and well-being. Finally, the parameter values used in this study were mainly obtained from literature and theoretical deduction, and the model is a theoretical simulation with no available large-scale survey data, so the accuracy of the values is insufficient, and future studies can calibrate these values with longitudinal data. This research selected a median value for the urban–rural disparity coefficient; however, this metric fluctuates across different areas, being relatively lower in prosperous eastern provinces and higher in western ones. Subsequent investigations should determine the specific urban–rural disparity factor based on local conditions and conduct contrasting simulations across diverse zones to investigate the regional variations in the underlying mechanisms through which digital divides impact the physical and mental health of older adults

## Data Availability

The original contributions presented in the study are included in the article/supplementary material, further inquiries can be directed to the corresponding author/s.
